# Complete genome sequence of human astrovirus genotype 6

**DOI:** 10.1186/1743-422X-7-29

**Published:** 2010-02-08

**Authors:** Li Guo, Richard Gonzalez, Wei Wang, Yongjun Li, Gláucia Paranhos-Baccalà, Guy Vernet, Jianwei Wang

**Affiliations:** 1 Dr. Christophe Mérieux Laboratory, IPB, CAMS- Fondation Mérieux and State Key Laboratory of Molecular Virology and Genetic Engineering, Institute of Pathogen Biology (IPB), Chinese Academy of Medical Sciences (CAMS), Beijing 100730, PR China; 2 Fondation Mérieux, 69365 Lyon, France

## Abstract

**Background:**

Human astroviruses (HAstVs) are one of the important causes of acute gastroenteritis in children. Currently, eight HAstV genotypes have been identified and all but two (HAstV-6 and HAstV-7) have been fully sequenced. We here sequenced and analyzed the complete genome of a HAstV-6 strain (192-BJ07), which was identified in Beijing, China.

**Results:**

The genome of 192-BJ07 consists of 6745 nucleotides. The 192-BJ07 strain displays a 77.2-78.0% nucleotide sequence identity with other HAstV genotypes and exhibits amino acid sequence identities of 86.5-87.4%, 94.2-95.1%, and 65.5-74.8% in the ORF1a, ORF1b, and ORF2 regions, respectively. Homological analysis of ORF2 shows that 192-BJ07 is 96.3% identical to the documented HAstV-6 strain. Further, phylogenetic analysis indicates that different genomic regions are likely undergoing different evolutionary and selective pressures. No recombination event was observed in HAstV-6 in this study.

**Conclusion:**

The completely sequenced and characterized genome of HAstV-6 (192-BJ07) provides further insight into the genetics of astroviruses and aids in the surveillance and control of HAstV gastroenteritis.

## Background

Human astroviruses (HAstVs) are one of the most common causes of acute gastroenteritis in children worldwide [1-3]. HAstV was first identified during an outbreak of gastroenteritis among hospitalized infants in 1975 [4]. Its name is derived from its distinctive star-shaped appearance under the electron microscopy (EM). Molecular analyses indicate that HAstVs are non-enveloped viruses with a 6-8 kb single-stranded, positive-sense RNA genome consisting of three overlapping open reading frames (ORFs)--ORF1a, ORF1b and ORF2--as well as the 5'- and 3' nontranslated regions (NTRs) [5]. ORF 1a encodes a serine protease; ORF 1b encodes an RNA dependent polymerase; and ORF 2 encodes a capsid precursor protein [5].

HAstVs have been grouped into eight known serotypes (HAstV-1 through HAstV-8) based on their reactivity to polyclonal antibodies and on analysis by immunofluorescence assays, neutralization assays, and immunoelectron microscopy (IEM) [5-7]. Phylogenetic analyses of the HAstV nucleotide sequence have defined eight genotypes, and further studies have indicated a strong correlation between the genotypes and serotypes [8]. As such, genotypes are frequently applied to type HAstVs.

Genomic characterization studies are important to the understanding of the origin, molecular evolution, and phylogenetic relationships among HAstV genotypes. The full-length genome sequence for a HAstV (HAstV-2) was first determined in 1993 [9]. Subsequently, the complete genomic sequences of five more genotypes (HAstV-1, HAstV-3, HAstV-4, HAstV-5, and HAstV-8) were reported [9-12]. Because the dominant, disease-causing HAstV type and strain often fluctuate with time and geographic location, it is critical that we characterize the complete genomic sequences of all known genotypes in order to better control and prevent future epidemics [13]. Limited sequence information for HAstV genotype 6 is available. Only a partial genome sequence has been reported [14,15], even though this genotype has been identified as one cause of sporadic or large scale outbreaks of acute gastroenteritis worldwide [16,17].

In 2007, we identified a case of HAstV-6 infection in Beijing, China, suggesting that this strain might be more epidemiologically relevant than previously recognized [18]. Here we sequenced and analyzed the complete genomic sequence of this HAstV-6 192-BJ07 strain, and describe its genetic characteristics by comparing its sequence with other known HAstV genotypes. The characterization of HAstV-6 by whole genome sequencing provides critical insight into the genetics of this virus as well as valuable information for the control and prevention of HAstV-induced gastroenteritis.

## Results

### Genome organization

**Figure 1:**
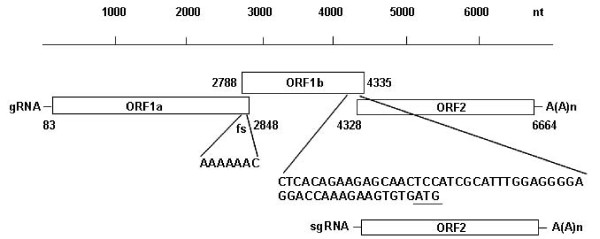
**Genome organization of the HAstV-6 192-BJ07 strain**. The ORFs were predicted as described in the *Materials and Methods *section. Genomic (gRNA) and subgenomic (sgRNA) RNA with open reading frame (ORF) ORF1a, ORF1b, and ORF2 are represented as boxes. The nucleotide sequences represent highly conserved sequences present in the frameshift signal (fs, AAAAAAC) and in the 52 nt of ORF1b/ORF2 junction.

Complete genome sequencing of HAstV-6 (192-BJ07) was performed from a stool sample that tested positive for HAstV-6 RNA fragments by RT-PCR. Starting with the cloning of ORF2, the entire sequence of the viral genome was obtained by a step-wise amplification strategy through 5'- and 3'-RACE. The full-length genomic RNA of 192-BJ07 is 6745 nucleotides (nt) in length, excluding a poly-A tail at the 3' end. HAstV-6 (192-BJ07) has the same genome organization as other known HAstV genotypes. It has a 5'-NTR of 82 nt, a 3'-NTR of 81 nt, and three overlapping major ORFs: ORF1a (2766 nt), ORF1b (1548 nt), and ORF2 (2337 nt). Details of the predicted genome organization of 192-BJ07 are shown in Fig. 1.

### ORF analysis

The sequence of the HAstV-6 192-BJ07 strain displayed similarity to those of other known HAstV genotypes. ORF1a of the HAstV-6 192-BJ07 strain shared 79.0%-79.9% nucleotide identity and 86.5-87.4% amino acid identity with those of genotypes 1 through 5 and with genotype 8. Two mutation sites were found at amino acids 757 and 758 in 192-BJ07 ORF1a, which result in the insertion of Arg and Lys.

Unlike ORF 1a, ORF1b exhibited higher nucleotide (85.7%-87.5%) and amino acid (94.2-95.1%) identity with that of genotypes 1 through 5 and genotype 8 (Table 1), indicating that ORF1b is more conserved among the 192-BJ07 strain and the HAstV genotypes than ORF 1a.

**Table 1:** Sequence identity between HAstV-6 (192-BJ07) and other HAstV genotypes

	Percent identity							
	
	5'NTR	ORF 1a		ORF 1b		ORF 2		3'NTR
Genotype (Genbank accession numbers)	Nucleotide	Nucleotide	Amino acid	Nucleotide	Amino acid	Nucleotide	Amino acid	Nucleotide
HAstV-1 (L23513)	76.5	79.3	87.1	86.4	94.8	71.3	71.4	98.8
HAstV-2 (L13745)	42.7	79.4	87.3	86.0	94.2	69.9	67.5	95.1
HAstV-3 (AF141381)	58.5	79.0	86.6	85.7	94.6	72.6	72.8	96.3
HAstV-4 (AY720891)	76.8	79.9	87.0	86.5	95.1	62.4	65.6	92.6
HAstV-5 (DQ028633)	57.3	79.2	86.5	87.5	94.6	72.1	74.8	96.3
HAstV-6 (Z46658)	NA	NA	NA	NA	NA	96.3	95.9	NA
HAstV-7 (Y08632)	NA	NA	NA	NA	NA	72.4	72.3	NA
HAstV-8 (AF260508)	57.8	79.8	87.4	86.0	94.8	70.9	72.8	97.5

NA: Not available.

Pairwise comparisons of the nucleotide and amino acid sequences of the ORF2 region showed that 192-BJ07 shares relatively low identity with other known HAstV genotypes in this region (62.4-72.6% nucleotide identity, and 65.5-74.8% amino acid identity; Table 1). However, 192-BJ07 exhibited high identity--96.3% nucleotide identity and 95.9% amino acid identity--with the documented sequence of the HAstV-6 strain (GenBank accession number Z46658 and Table 1). Structural predictions of ORF2 indicated that there are three highly conserved amino acid residues that can be cleaved to yield proteins with different sizes: Lys 71 for a 79-kDa protein, Arg 361 for VP29 and Arg 395 for VP26 [19].

**Figure 2:**
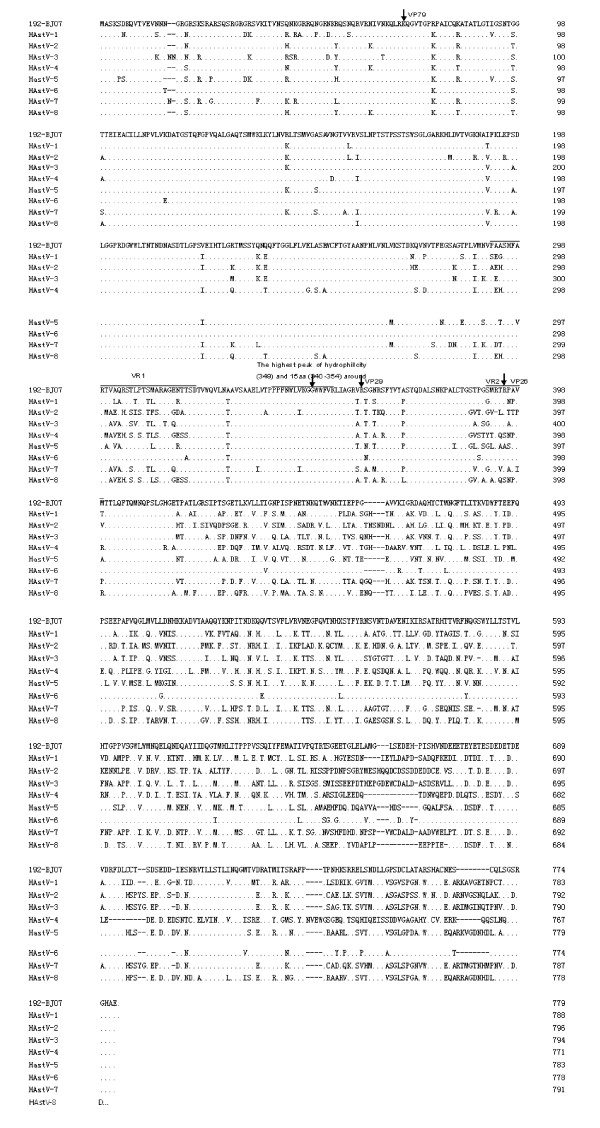
**Alignment of the complete ORF2 amino acid sequence of 192-BJ07 with other HAstV genotypes**. The alignment analysis was performed using the MegAlign programs in the DNAStar software package. Variable regions VR1 and VR2 are underlined. Dots indicate amino acid sequence identities, and a dash denotes a deletion. The single-letter amino acid code is used. GenBank accession numbers: HAstV-1: L23513; HAstV-2: L13745; HAstV-3: AF141381; HAstV-4: AY720891; HAstV-5: DQ028633; HAstV-6: Z46658; HAstV-7: Y08632; HAstV-8: AF260508.

Alignment of the 192-BJ07 ORF2 sequence with the seven other HAstV genotypes showed that the conserved domain (N-terminal 415 amino acids) shares 85.8-89.6% identity (Fig. 2); whereas the region from amino acid 416 to the carboxyl-terminus, i.e. the variable region, showed higher levels of variation, with amino acid sequence identities of only 41-58% (Fig. 2). Compared with other HAstV genotypes, the 192-BJ07 strain displayed 25-57.5% variation in VR1 (aa 292-319) and 30.8-92.3% in VR2 (aa 386-399) [20] (Fig. 2). These results are consistent with those described in a previous report on HAstV-8 by Méndez-Toss et al [20,21].

### Non-coding region analysis

**Figure 3:**
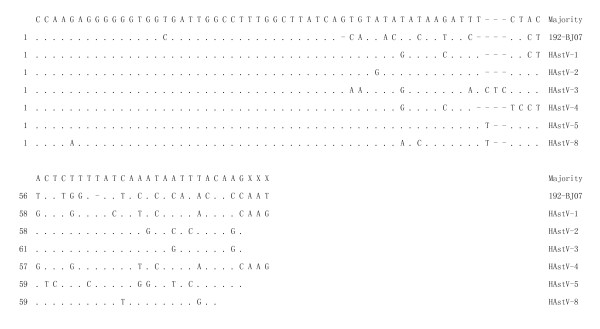
**Alignment analysis of the 5'-NTR among HAstV genotypes**. The alignment analysis was performed using the MegAlign programs in the DNAStar software package. Dots indicate nucleotide sequence identities; a dash denotes a deletion. GenBank accession numbers: HAstV-1: L23513; HAstV-2: L13745; HAsstV-3: AF141381; HAstV-4: AY720891; HAstV-5: DQ028633; HAstV-8: AF260508.

HAstV genotypes typically contain 80 to 85 nt in the 5'-NTR. The region shows the highest nucleotide variation (23.2-57.3%) among the five regions of the viral genome (5'-NTR, ORF1a, ORF1b, ORF2, and 3'-NTR) (Table 1). However, the 37 nt of the very 5' end of the viral genome are highly conserved (Fig. 3). Secondary structural predictions indicate that the HAstV-6 192-BJ07 strain has three stem-loop structures in the 5'-NTR. However, because there is a high level sequence variability within the region of nt 38-85 (Fig. 3), the consensus of the stem-loop structures is very low among HAstV genotypes (data not shown).

In contrast, we found that the nucleotide identities of the 3'-NTR sequences are as high as 92.6-98.8% compared with other known HAstV genotypes. However, the sequence variability within this region also results in secondary structure disparities of the 3'-NTR between HAstV genotypes (data not shown).

It has been recognized that HAstV RNA has a cis-acting element [ribosomal frameshifting heptamer sequence (AAAAAAC)] followed by a stem-loop structure in the ORF1a/1b junction region [22]. The 192-BJ07 strain also has such a shifty heptamer sequence and a similar stem-loop structure based on analysis with RNAstructure 4.5 software. This conservation may reflect the importance of such structures for translational regulation [22].

The ORF1b/ORF2 junction has been regarded as a regulatory element of the sub-genomic RNA (sgRNA) [23]. The alignment analysis of 52 nt at the ORF1b/ORF2 junction revealed that 192-BJ07 has a very high identity (98.4-100%) with other HAstV genotypes, consistent with a previous report [24].

### Phylogenetic analysis

**Figure 4:**
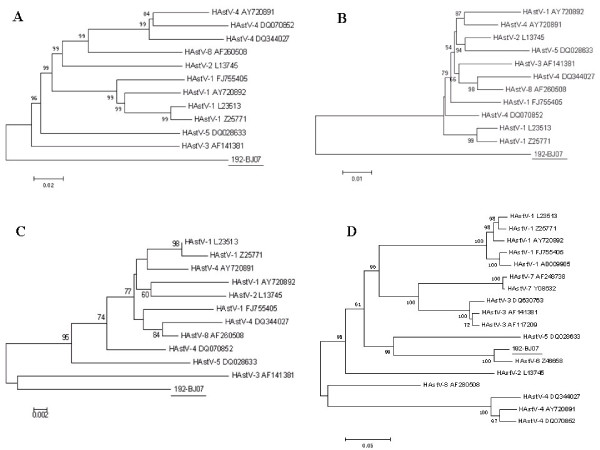
**Phylogenetic analysis of the 192-BJ07 strain based on full-length genome sequencing**. The phylogenetic trees were generated using MEGA 4.0 software based on nucleotide sequences of full-length genomes (A), and on amino acid sequences of the ORF1a (B), ORF1b (C), and ORF2 (D) regions.

Nucleotide alignment analysis of whole genome sequences showed that the identities between the HAstV-6 192-BJ07 strain and the corresponding sequences of HAstV-1, -2, -3, -4, -5 and -8 were 77.2-78.0%. We found that the evolutionary relationships among HAstV genotypes were divergent when phylogenetic analyses were performed at the level of complete genome sequence or at the level of individual proteins. The phylogenetic analysis of the whole genome sequence and the ORF1a region indicates that the HAstV-6 192-BJ07 strain is a significant outlier on the phylogenetic tree compared to the other HAstV genotypes (Fig. 4A and 4B). In the region of ORF1b, the HAstV-6 192-BJ07 strain and HAstV-3 branch out earlier (Fig. 4C); while in the region of ORF2, HAstV-8 and HAstV-4 share the common ancestor role of other HAstV genotypes (Fig. 4D).

### Recombination analysis

**Figure 5:**
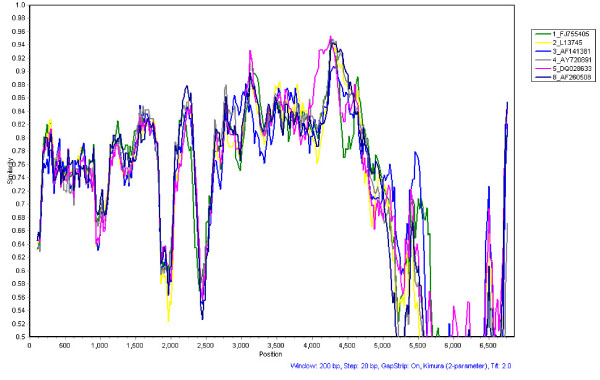
**Similarity analysis of HAstVs based on complete genome sequences**. Similarity plots were created using the SimPlot software version 3.5.1. Data are shown as percentages of the identities to the putative parental strains. The numbers 1, 2, 3, 4, 5, and 8 represent complete genomes of HAstV-1 (GenBank accession number L23513), HAstV-2 (GenBank accession number L13745), HAstV-3(GenBank accession number AF141381), HAstV-4 (GenBank accession number AY720891), HAstV-5 (GenBank accession number DQ028633) and HAstV-8 (GenBank accession number AF260508), respectively.

To determine whether there were recombination events between the 192-BJ07 strain and other known HAstV genotypes, we analyzed the similarities at the genome level. A similarity plot comparing the nucleotide sequences of HAstV-6 192-BJ07 and HAstV-1, -2, -3, -4, -5, and -8 is shown in Fig. 5. The sequence identities of the 192-BJ07 ORF1b region with the other genotypes are higher than those of ORF1a and ORF2. This may indicate conservation of ORF1b among HAstVs. However, no recombination break points were detected between the query sequence (HAstV-6 192-BJ07) and the reference sequences using the SimPlot program. Thus, there is no clear evidence of genetic recombination between 192-BJ07 and other HAstV genotypes.

## Discussion

In this study, we report the whole genome sequence of HAstV-6 based on a strain (192-BJ07) identified in an etiological investigation of viral gastroenteritis in Beijing [18]. The sequence analysis shows that the 192-BJ07 strain has a typical astrovirus genome organization with three ORFs (ORF1a, ORF1b, and ORF2), an 80-85 nt 5'-NTR, and an 80-85 nt 3'-NTR. Phylogenetic and homological analyses of the ORF2 regions indicate that the 192-BJ07 strain genome possesses a 95.9% amino acid identity to the documented HAstV-6 strain (GenBank accession number Z46658), but a <75% amino acid identity to other HAstV genotypes.

Consistent with previous reports of other HAstV genotypes, our results also show the existence of three potential cleavage sites at Lys 71, Arg 361, and Arg 395 in HAstV6 ORF2 [3,19,20]. It is thought that the cleavage at Lys 71 leads to the generation of the 79-kDa capsid protein [19]. The 79-kDa capsid protein can be converted into three smaller peptides--VP34, VP29, and VP26--and leads to an enhancement of HAstV infectivity [19]. Our observations support the critical role of these three amino acid residues in HAstV replication and pathogenesis.

In our study, we found two insertional mutations, Arg 757 and Lys 758, in ORF1a. How these hydrophilic amino acids contribute to the characteristic/function of the virus is unknown at present and needs to be addressed in further functional studies.

Our phylogenetic analysis suggests that HAstV-6 may be an ancestor of other HAstV genotypes as shown by the phylogenetic analysis of the whole genome sequence (Fig. 4A). This observation was further supported by the phylogenetic analysis of the ORF1a protein region (Fig. 4B). Moreover, detailed analysis of all genotype ORF1b amino acid sequences indicates that HAstV-6 and HAstV-3 may have functioned as the common ancestor of other HAstV genotypes (Fig. 4C). However, the analysis of HAstVs ORF2 suggests that HAstV-8 and HAstV-4 may have been the common ancestor of other HAstV genotypes (Fig. 4D). Different evolutionary and selective pressures in different HAstV genomic regions may be responsible for this discrepancy of the evolutionary relationships [25].

The secondary structure predictions indicate that stem-loop structures are not conserved in the 5'- and 3'-NTRs of known HAstV genotype genomes. This difference may be responsible for the possible discrepancy at the replication and/or transcription level among HAstV genotypes. The fact that the 5'-end of the 5'-NTR and the 3'-NTR and the 52 nt region at the ORF1b/ORF2 junction are highly conserved points to their critical role in the interaction with the viral replicative or transcriptive machinery. The variation in the 3'-end of 5'-NTR may influence the efficiency of viral genome replication or transcription, resulting in a difference in replication ability or virulence among different genotypes or strains [5].

The -1 ribosomal frameshifting is critical for the translation of the astrovirus genome [22]. The -1 ribosomal frameshifting requires two cis-acting signals: a shifty heptamer sequence (AAAAAAC) and a potential stem-loop structure [10,26]. This study showed that the HAstV-6 192-BJ07 strain also has such cis-acting elements, and further demonstrates the conservation of such elements among HAstV genotypes [5].

At present, the mechanism of HAstVs' variations is unclear. One study has indicated that recombination may be responsible for HAstVs' variation [24]. However, current studies have not broadly established the role of recombination in HAstV variation [25,27]. In agreement with most reports, we found no clear evidence of recombination between the 192-BJ07 strain and other HAstV genotypes based on similarity plot analysis. Diversification of the HAstV amino sequences may be attributed to accumulated single nucleotide mutations. This mechanism is similar to the antigen drift in other viruses, such as in influenza viruses [28,29], which could lead to HAstVs escaping from existing host immunities and could result in the emergence of a new epidemic HAstV strain [30]. Additional studies, such as large scale whole genome sequencing, are needed to address the evolutionary patterns of HAstVs.

## Conclusion

We have sequenced and characterized the complete genome of HAstV-6 (192-BJ07). This sequence will provide insight into the genetics of astroviruses, broaden our understanding of their properties, and inform surveillance and control of HAstV gastroenteritis around the world.

## Methods

### RNA extraction

A stool sample (termed 192-BJ07) that tested positive for HAstV-6 by RT-PCR was collected from a 2-year old boy who visited the Beijing Children's Hospital with acute diarrhea in 2007 [18]. Viral RNA was extracted from the stool supernatant using Trizol reagent (Invitrogen, Carlsbad, CA) according to the manufacturer's instructions.

### ORF2 amplification

The primers ORF2-F (5'-atggctagcaagtctgacaagcagg-3') and ORF2-R (5'-gaagctgtaccctcgatcctactc-3') targeting ORF2 of 192-BJ07 were designed based on the only available HAstV-6 sequence in GenBank (GenBank accession number Z46658). For reverse transcription (RT) reactions, cDNA was generated with the SuperScript™ III RT kit (Invitrogen, Carlsbad, CA) using a random primer (Takara, Dalian, China) as described in the manufacturer's protocol. The PCR reaction was performed as follows: 94°C for 3 minutes, 35 cycles of amplification (94°C for 30 seconds; 50°C for 30 seconds; and 72°C for 3 minutes), and a final 10 minutes extension at 72°C. The PCR products were analyzed by 1.0% agarose gel electrophoresis and stained with ethidium bromide.

### Genome amplification and sequencing

Rapid amplification of cDNA end (RACE) reactions were performed to obtain the entire sequence of the viral genome by using the 5'- and 3'-RACE System for Rapid Amplification of cDNA Ends kit (Invitrogen, Carlsbad, CA) according to the manufacturer's protocol. The ORF2 sequence obtained above was used as the starting point for the amplification. PCR-amplified products were cloned into the pMD18-T vector (TaKaRa, Dalian, China) and were introduced into chemically competent *E. coli *DH5α cells. The plasmid DNA was sequenced using an ABI3730 DNA Analyzer (Applied Biosystems). The complete genome sequence of HAstV-6 has been deposited in GenBank (GenBank Accession number GQ495608).

### ORF prediction and RNA structure analysis

ORF1a and ORF2 were predicted for HAstV-6 192-BJ07 using the DNAStar ORF search program. ORF1b was predicted based on the "shifty"' heptanucleotide (AAAAAAC) that occurs in other HAstVs [9]. RNA secondary structures were evaluated using RNAstructure 4.5 software.

### Phylogenetic analysis

The MegAlign programs in the DNAStar software package were used to perform multiple sequence alignments. HAstV phylogenies with 1000 bootstrap replicates were created using the neighbor-joining method and the Kimura two-parameter model with the MEGA software version 4.0 [31].

### Similarity analysis

SimPlot software version 3.5.1 [32] was used to analyze the relationships among the aligned HAstV genome sequences. The complete genome sequences of 192-BJ07, HAstV-1 (GenBank accession numbers L23513), HAstV-2 (GenBank accession number L13745), HAstV-3 (GenBank accession number AF141381), HAstV-4 (GenBank accession numbers AY720891), HAstV-5 (GenBank accession number DQ028633), and HAstV-8 (GenBank accession number AF260508) were first aligned by using Clustal W of the MEGA 4 program, and then 192-BJ07 was chosen as the query sequence for the similarity analysis. Similarity was calculated in each window of 200 bp using the Kimura two-parameter method.

## Competing interests

The authors declare that they have no competing interests.

## Authors' contributions

JW, RG, GPB, and GV conceived the study. LG and JW designed the experiments. LG and WW carried out the experiments and analysis. YL participated in sequence analysis. LG, RG, and JW wrote the manuscript. All authors critically read and approved the final manuscript.
